# Renal Clear Cell Carcinoma Masquerading As Brain Arteriovenous Malformation: A Case Report and Review of the Literature

**DOI:** 10.7759/cureus.75447

**Published:** 2024-12-10

**Authors:** Yew Weng Fong, Sean Himel, Cristina Cernei, Kathreena Kurian, Kanjilal Banhisikha, James Wareham, Mario Teo

**Affiliations:** 1 Division of Neurological Surgery, Department of Surgery, Cathay General Hospital, Taipei City, TWN; 2 Neurosurgery, Southmead Hospital, North Bristol NHS, Bristol, GBR; 3 Neurosurgery, Department of Neurosurgery, University of Tennessee Health Science Center, Memphis, USA; 4 Neuropathology, Southmead Hospital, North Bristol NHS, Bristol, GBR; 5 Neuroradiology, Southmead Hospital, North Bristol NHS, Bristol, GBR

**Keywords:** brain arteriovenous malformation, brain metastasis, early arteriovenous shunting, intracerebral hemorrhage, renal clear cell carcinoma

## Abstract

Cerebral arteriovenous malformations (AVMs) are tangles of abnormal vessels with early arteriovenous (AV) shunting that can lead to intracerebral hemorrhage, seizures, neurologic deficit, or headache. To date, only a few cases of carcinomas metastasizing to pre-existing cerebral AVMs have been reported in the literature. However, renal clear cell carcinoma (RCC) brain metastases that exhibit early AV shunting, where AVM pathology is not present, are extremely rare. We report a very rare case of early AV shunting phenomenon in RCC brain metastasis. This is a case of a 57-year-old man with no prior medical history who presented to the emergency department with an acute onset headache and speech difficulties. Contrast-enhanced computed tomography of the head revealed a left temporal intracerebral hemorrhage with a vascular nidus adjacent to the hematoma. A diagnostic cerebral angiogram (DSA) showed a Spetzler-Martin grade II AVM in the left para-Sylvian region, supplied by middle cerebral arteries with a superficial draining vein. Gadolinium-enhanced brain MRI revealed evolving methemoglobin and a small compact nidus adjacent to the hematoma in the distal left Sylvian fissure. The patient was stable until day 17 after hospital admission, when he deteriorated due to an interval repeat hemorrhage, requiring surgical excision of the AVM and the hematoma. Postoperative DSA revealed no residual shunting and complete resection of the AVM. However, there was a further re-bleed postoperatively, necessitating a second surgery. Pathology revealed an RCC without any specific pathological features indicative of an AVM. The patient recovered to his baseline but died in less than a year due to the progression of metastatic disease. In conclusion, RCC can exhibit an early AV shunting phenomenon, with the presence of enlarged, tortuous, pathologic nidal-like tumor-feeding vessels, which can mimic an AVM, which is extremely rare. This phenomenon poses significant treatment challenges, as the treatment goals are completely different for AVMs and metastatic disease.

## Introduction

Intracerebral hemorrhage (ICH) is a devastating type of stroke accounting for 6.5% to 19.6% of strokes worldwide with high mortality [1]. Underlying etiologies include chronic hypertension, cerebral amyloid angiopathy, vascular malformations, neoplastic lesions, hemorrhagic transformation of an ischemia stroke, and venous thrombosis [2]. Certain distinctive hemorrhage patterns lead physicians to obtain advanced imaging studies to achieve a more complete diagnosis and therefore to formulate the most effective treatment strategies. In younger patients and when ICHs are in atypical locations of the brain, further vascular imaging studies such as computed tomography angiography (CTA) and magnetic resonance angiography (MRA) are warranted to assess for causative underlying pathologies.

Arteriovenous malformations (AVMs) contain vascular niduses with early arteriovenous (AV) shunting without intervening capillary beds. AVMs have the propensity to rupture as the walls of these arterial feeders, niduses, arterialized veins, and flow-related aneurysms weaken [3]. With an incidence of 0.94 per 100,000 people, AVMs have an approximate 2.3% risk of annual rupture. The mortality rate after AVM rupture is approximately 10%, with a 30-50% rate of morbidity [4].

The goal of treatment is to eliminate the risk of future hemorrhage, which can only be achieved with complete resection of the nidus. Microsurgical resection offers the highest obliteration rate with relatively low complication rates when performed by experienced neurosurgeons signifying it as the gold standard treatment for low-grade AVMs [5]. We present the case of a clinical and radiological AVM mimic that turned out to be a renal clear cell carcinoma (RCC) brain metastasis.

## Case presentation

**Figure 1:**
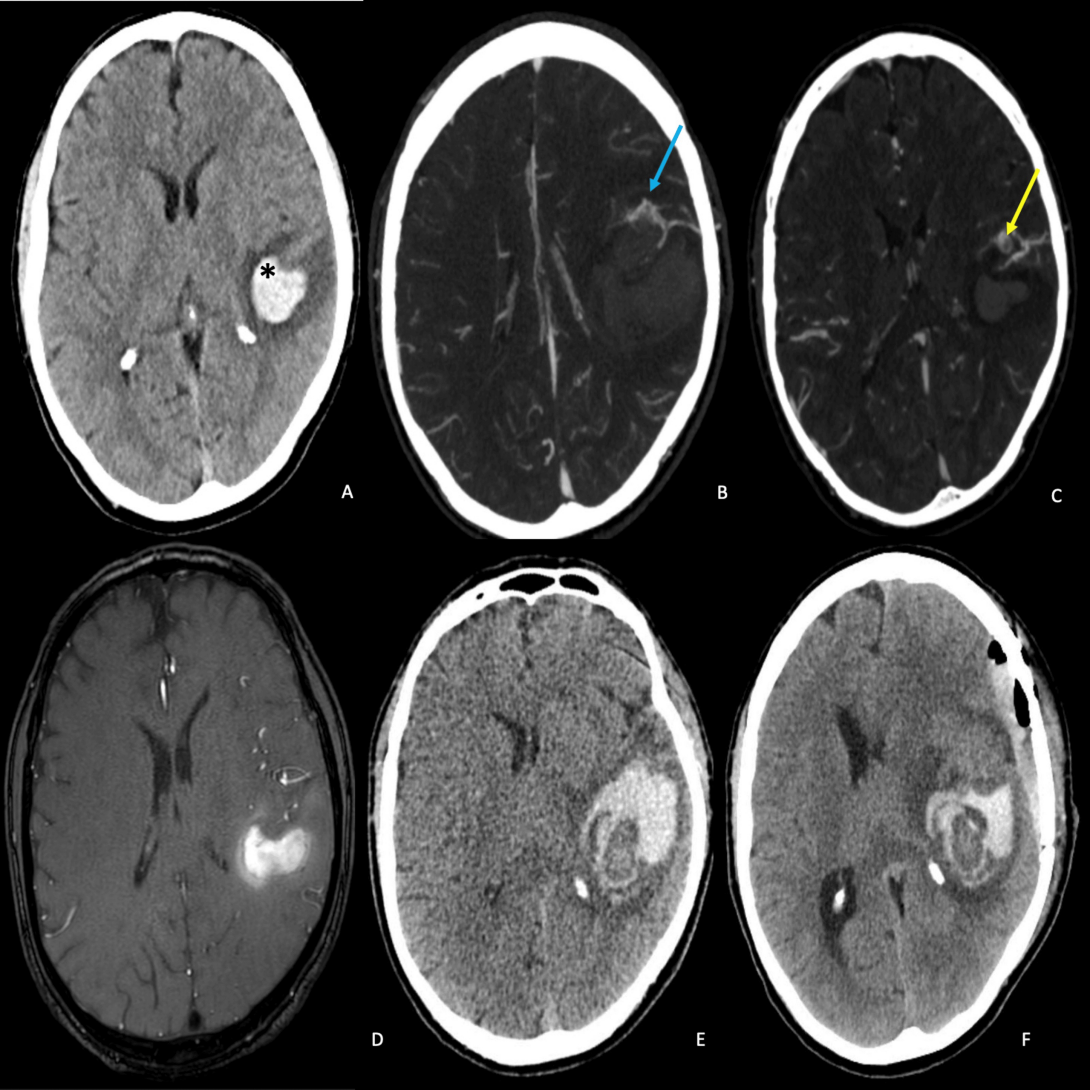
Serial imaging studies showing the disease progression. (A) CT brain (axial view) showed a hematoma (*) in the left parietal region near the Sylvian fissure. (B, C) Brain CTA revealed a nidus-like lesion (Blue arrow), a rounded structure (Yellow arrow) with prominent cortical vessels in this region, suggestive of an arteriovenous malformation (AVM). (D) Gadolinium-enhanced brain MRI (axial view) showed evolving methemoglobin with mild perifocal edematous changes, consistent with evolving methemoglobin. (E) Brain CT was repeated on day 17 after the initial bleed showing a recurrent bleed. (F) Further rebleeding in the surgical cavity was noted, along with postoperative changes, including a mild subdural residual hematoma and pneumocephalus.

**Figure 2:**
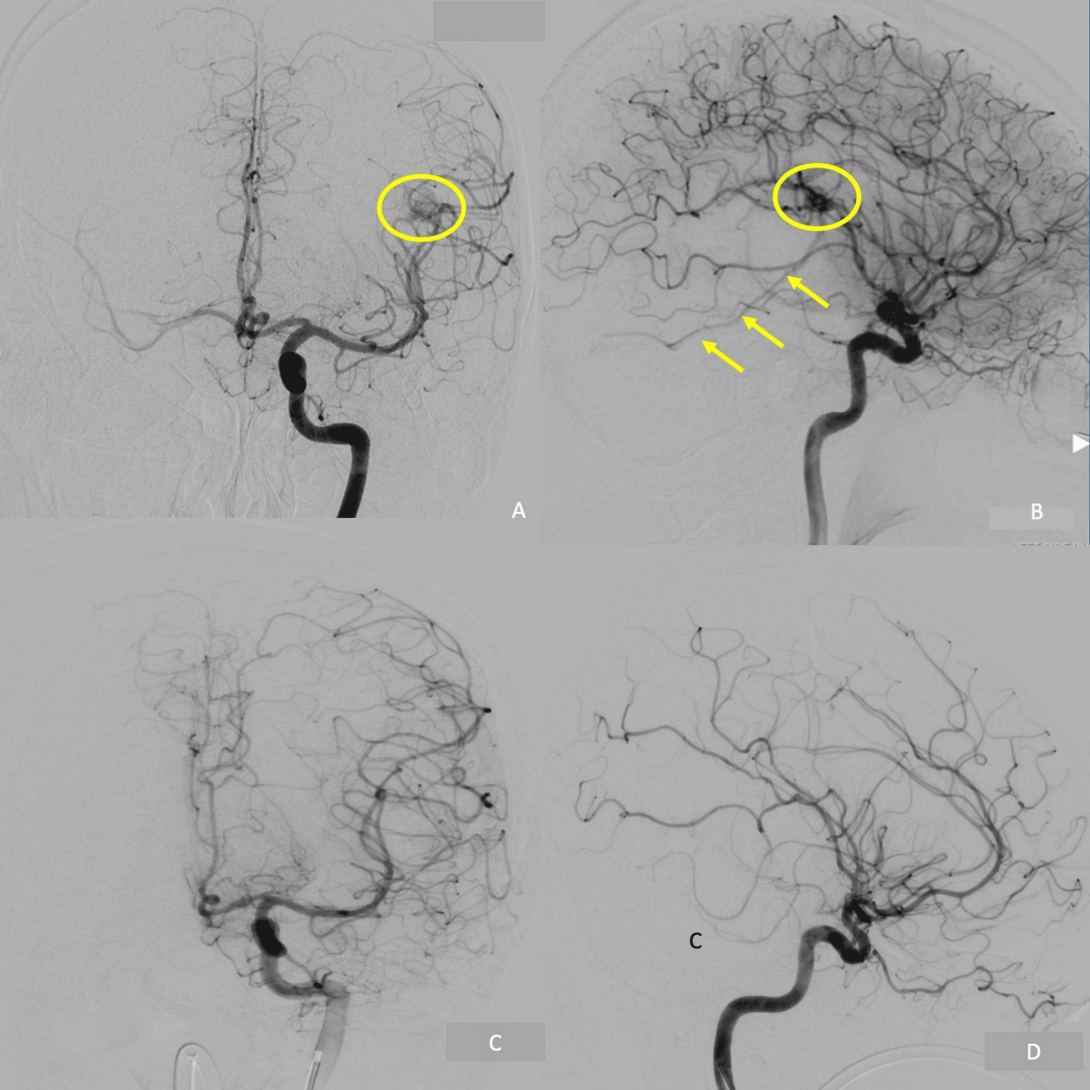
The diagnostic angiography of the right internal carotid pre- and post-surgical resection. Anteroposterior view (A) and lateral view (B) of the pre-operative DSA of the right internal carotid artery showed the AVM nidus (Yellow circle) supplied by middle cerebral arteries with an early draining into the vein of Labbe (Yellow arrows); while (C, D) demonstrated the AVM nidus and early AV shunt were no longer visible. AVM: arteriovenous malformation; DSA: diagnostic cerebral angiogram

A 57-year-old man with no significant past medical history presented to the emergency department with an acute onset headache localized to the tempo-parietal region and speech difficulties. On examination, he was aphasic but was fully conscious, oriented to person, time, and place, and obeying verbal commands. He was otherwise neurologically intact. Laboratory studies revealed no significant abnormalities. Non-contrast CT and CTA of the head showed a left posterior temporal ICH near Wernicke’s area and enlarged, tortuous blood vessels over the left cerebral hemisphere anterior to the hematoma (Figures 1A-1C). A contrast-enhanced MRI revealed evolving methemoglobin with mild perifocal edema in the distal left Sylvian fissure (Figure 1D). A diagnostic cerebral angiogram (DSA) showed a Spetzler-Martin grade II AVM nidus measuring 10 mm, supplied by middle cerebral artery (MCA) feeders centered at the M2/3 junction, with superficial drainage to the vein of Labbe (Figures 2A-2B).

**Figure 3:**
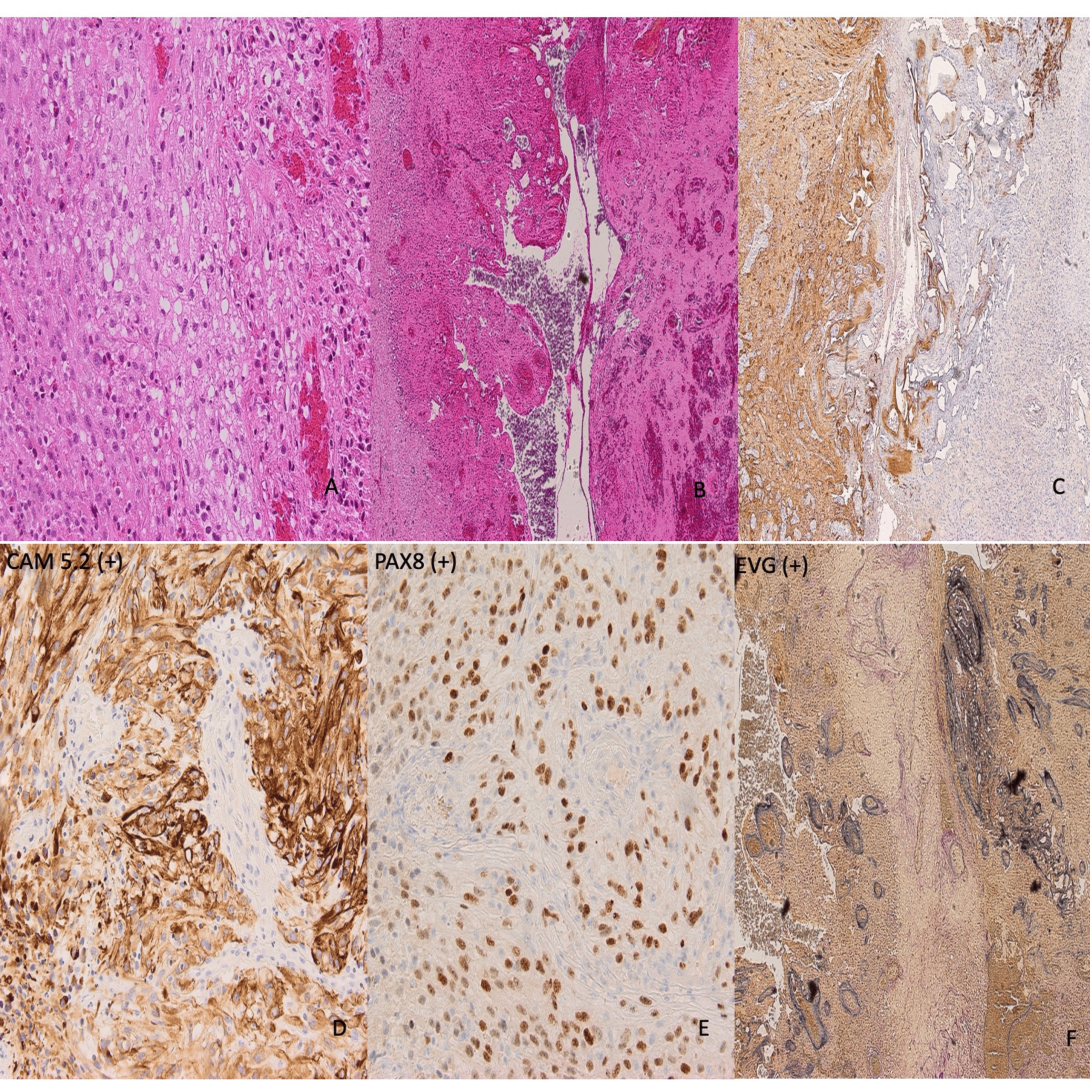
The photomicrographs of the surgical pathology specimens. A)The photomicrograph of the specimen showed pleomorphic metastatic cancer cells with clear cytoplasm. (B) The left side displays metastatic tumor, while the right side shows reactive glial tissue. (C) GFAP is positive in the left-sided gliotic parenchyma and negative in the right-sided neoplastic cells. (D) CAM 5.2 is positive in neoplastic cells, (E) PAX8 (a renal cell carcinoma marker) is positive in the neoplastic cells. (F) EVG is stained positively in abnormal vessels. EVG: Verhoeff-von Gieson

**Figure 4:**
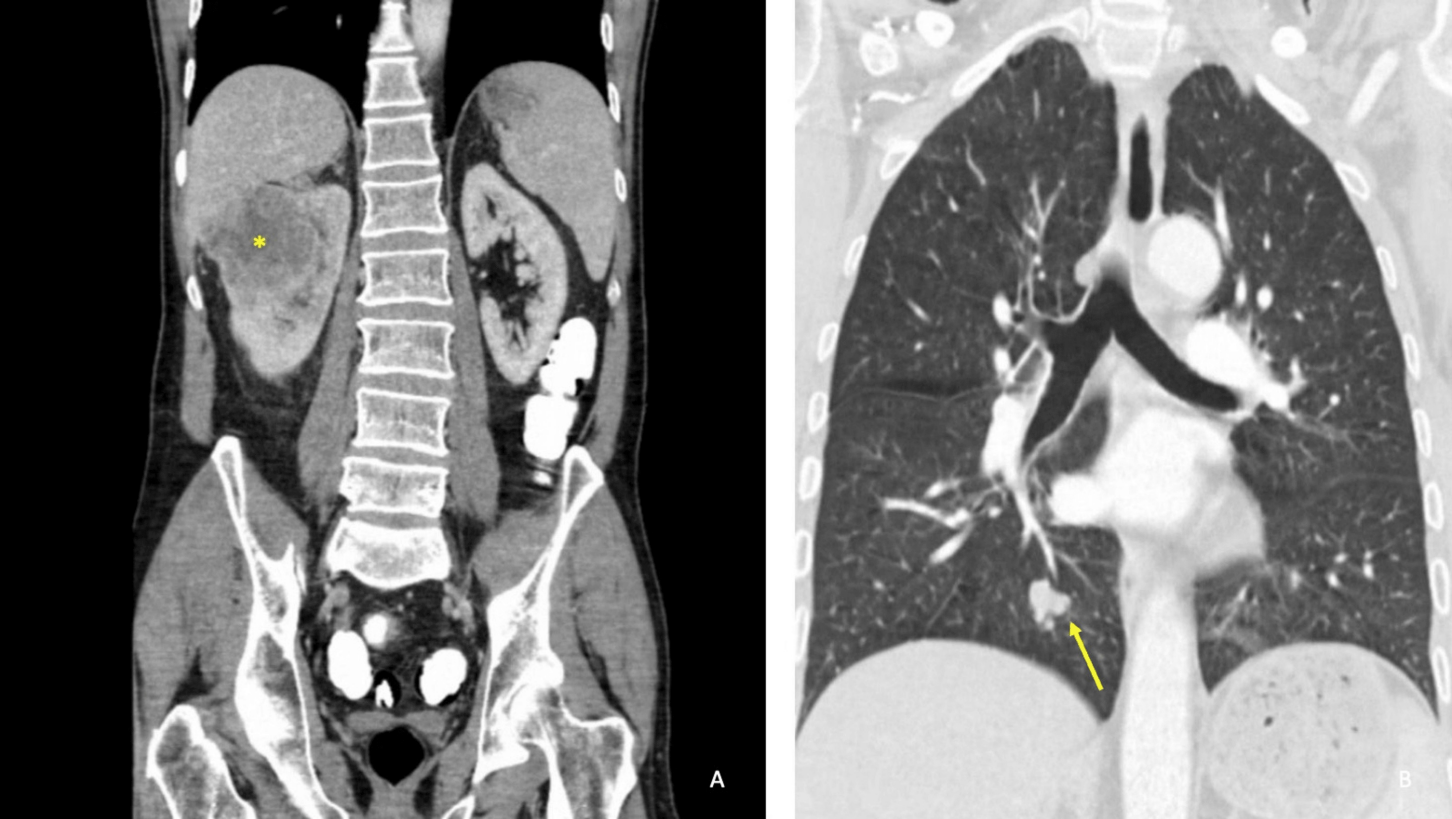
Computed tomography scan of the abdominopelvic (A) and chest (B) region. (A) Coronal CT image of the abdomen showing a renal tumor (*) located at the right upper pole, measuring 7 cm. (B) Coronal CT image of the chest showing lung metastasis (Yellow arrow).

Due to the eloquent location of the ICH and suspected AVM, clinical discussions centered around allowing the patient to recover from the hemorrhage followed by treatment (radiosurgery versus microsurgical resection) in a delayed fashion. Unfortunately, the patient experienced a second ICH on hospital day 17 (Figure 1E). After a neurovascular multidisciplinary team discussion, a neuro-navigation-guided craniotomy for hematoma evacuation and microsurgical resection of the lesion was offered to the patient and his family after a detailed explanation of the risks, benefits, indications, and alternatives. The patient and his family consented to the procedure. The operation was uneventful, however, on the postoperative CT scan the following day, it was noted that there was further ICH in the surgical cavity, necessitating an emergent repeat surgery for hematoma evacuation (Figure 1F). A DSA was repeated; however, no residual AVM was revealed (Figures 2C-2D). Histopathological examination of the pathology specimen obtained during the original operation revealed neoplastic cells stained positively for GFAP, CAM5.2, and PAX8 indicating metastatic renal cell carcinoma (Figure 3). The Verhoeff-von Gieson (EVG) stain showed accompanying abnormal distended blood vessels over the tumor which contain elastic laminae (Figure 3F). These findings were consistent with hemorrhagic metastatic clear cell carcinoma. Whole body CT scans later confirmed a renal cell carcinoma, measuring 7 cm, at the right upper pole, with lung metastasis (Figure 4).

Postoperatively, the patient improved to his presenting baseline. He was referred to the renal oncology team to commence systemic treatment. Due to ongoing metastatic disease progression, he died less than a year after the initial ICH presentation.

## Discussion

RCC accounts for less than 5% of all malignant tumors, which commonly metastasize to the bones, lungs, lymph nodes, and liver. Metastasis in the brain constitutes around 10-16%, while the 5-year cumulative incidence of approximately 9.8% [6]. These metastatic brain tumors carry a higher risk of spontaneous hemorrhage when patients have received tyrosine kinase inhibitors (TKI) medications, or radiation therapy [7]. In our case, the tumor hypervascularity and the highly vulnerable feeding vessels likely contributed to the intracranial hemorrhage [8]. Although RCC brain metastases hemorrhage easily, they do not often masquerade as an AVM.

ICH is a critical medical condition with a broad spectrum of etiologies that include hypertension, vascular malformations, or neoplastic lesions. It is crucial to recognize the underlying etiology that contributes to each ICH to tailor effective treatment strategies. Identifying the hemorrhagic intracranial neoplastic lesion is not always possible with non-contrast CT or CTA alone. They may bleed in atypical locations, have irregular shapes, present with multiple hemorrhagic foci, have a heterogenous hematoma appearance, have marginal enhancement, and have disproportionate perifocal edema [9]. MRI can be helpful in diagnosing underlying lesions; however, intra-axial hematomas often have heterogeneous features, and these vary over time. The transformation of these hematomas in the subacute to chronic phase may closely mimic tumor characteristics, and may also enhance over the periphery of the lesion with surrounding edema. AVMs generally present as hypointense on T1-weighted images (WI) and hyperintense with prominent flow voids on T2-WI [9]. Despite advanced imaging techniques, underlying tumors are often obscured by the surrounding hemorrhage [10]. Consequently, it can be difficult to discern whether the imaging findings represent a tumor, a tumor mimic, or simply a pure hematoma in one of its different phases of reabsorption.

Given our patient had no known risk factors and the hematoma was in an atypical location, the etiology of the spontaneous intraparenchymal hemorrhage was investigated. The CTA of the head revealed irregular vasculature with a nidal-like lesion that spatially coincide with the hematoma. Diagnostic cerebral angiography demonstrated an early AV shunt along with a tightly packed group of vessels that appeared to be a nidus, which led to the diagnosis of an AVM. We initially planned to closely observe the patient due to the lesion being a low-grade AVM (Spetzler-Martin-II: S1V0E1) located in an eloquent area with a likely long-term plan for radiosurgery. Before this plan could be enacted, the patient deteriorated with an interval bleed requiring the hematoma to be evacuated and the lesion removed. Subsequent pathological analysis revealed an unexpected diagnosis of metastatic RCC, with no evidence supporting the presence of an AVM.

Carcinomas with intracranial metastases to pre-existing true AVMs have been reported in the literature but are incredibly rare occurrences. To date, only eight cases have been reported as previously described. Kazama et al. summarized six reported cases of carcinomas that metastasized to the brain in the literature, of which three cases were carcinomas originating from lung tissue, two from testicular tissue, and one from breast origin [11]. Two additional cases were found to have metastasized to the cerebral AVM with carcinomas of renal [12] and lung tissue [13] origin. The ages of these cases range from 19 to 73 years old. Three of the eight cases were female. Presenting symptoms varied, which included intracranial hemorrhage, seizures, and headaches. Six of the AVMs were found located in the supratentorial region, one in the infratentorial region, and one in the pons.

It has been proposed that the hypervascularity of the AVM may have entrapped tumor emboli, therefore leading to a higher chance of hematogenous metastasis [11,14]. To our knowledge, it is exceptionally rare for an intracranial metastatic RCC to manifest with an AVM-like shunt. The absence of an abnormal vascular structure resembling an AVM on pathological examination, further accentuates the presence of an AVM-like phenomenon in RCC brain metastasis in our case, rather than the presence of two separate disease entities-a true AVM and a metastatic tumor lesion, as reported by previous literature. The tangled vessels identified in the imaging studies were likely tumor-supplying vessels. The imbalance of the proangiogenic vascular endothelial growth factor (VEGF), erythropoietin (EPO), platelet-derived growth factor (PDGF), and insulin-like growth factor 2 (IGF2) could contribute to the structural and functional abnormalities in blood vessels, which may lead to early AV shunting in cerebral neoplasms [15]. The phenomenon of AV shunting in metastatic brain tumors from RCC could have significant implications for both diagnostic processes and therapeutic approaches.

The treatment goals are significantly different for these two different diseases. The surgical management of metastatic brain tumors aims for a gross total resection (GTR) whenever feasible to improve patient outcome; while the management of a ruptured AVM is to eliminate future recurrent bleeding through surgical resection, stereotactic radiosurgery, endovascular treatment, conservative management, or a combination of these treatment options. For low-grade ruptured AVMs, active intervention including surgical resection of the nidus is often the treatment of choice to eliminate the future risk of recurrent bleeding but should be a tailored approach for each patient based on multidisciplinary input. In this case, we treated the patient with surgical resection of the suspected AVM lesion to remove all AV shunting to prevent future rebleeding. The pathology revealed a metastatic tumor with AVM-like shunting complicating the clinical landscape. If the lesion was known to be a hemorrhagic brain metastasis, then the timing of the surgery and overall surgical plan would likely have been different. Therefore, it is significant to understand the possibility of the presence of AV shunting in metastatic renal cell carcinoma which can lead to different treatment paradigms.

## Conclusions

To our knowledge, it is extremely rare for RCC with brain metastasis to display an early AV shunting phenomenon, accompanied by nidal-like tumor-feeding vessels mimicking an AVM, therefore leading to a completely different diagnosis. In this case, the initial imaging suggested an AVM when, in fact, it was a brain metastasis arising from RCC. Consequently, it is crucial to distinguish these two different disease entities, as their treatment goals vary significantly. We would be able to tailor a better treatment plan if this early shunting phenomenon in brain metastasis of RCC was recognized in advance, ultimately leading to better patient outcomes.
